# Antioxidant activities and fatty acid composition of wild grown myrtle (*Myrtus communis* L.) fruits

**DOI:** 10.4103/0973-1296.59960

**Published:** 2010-02-13

**Authors:** Sedat Serce, Sezai Ercisli, Memnune Sengul, Kazim Gunduz, Emine Orhan

**Affiliations:** *Agricultural Faculty, Department of Horticulture, Mustafa Kemal University, 31034 Antakya, Hatay, Turkey*; 1 *Agricultural Faculty, Department of Horticulture, Ataturk University, 25240 Erzurum, Turkey*

**Keywords:** Antioxidant activity, diphenylpicrylhydrazyl, Mediterranean, myrtle

## Abstract

The fruits of eight myrtles, *Myrtus communis* L. accessions from the Mediterranean region of Turkey were evaluated for their antioxidant activities and fatty acid contents. The antioxidant activities of the fruit extracts were determined by using 2,2-diphenyl-1-picrylhydrazyl (DPPH) and β-carotene-linoleic acid assays. The fatty acid contents of fruits were determined by using gas chromatography. The methanol extracts of fruits exhibited a high level of free radical scavenging activity. There was a wide range (74.51-91.65%) of antioxidant activity among the accessions in the β-carotene-linoleic acid assay. The amount of total phenolics (TP) was determined to be between 44.41-74.44 μg Gallic acid equivalent (GAE)/mg, on a dry weight basis. Oleic acid was the dominant fatty acid (67.07%), followed by palmitic (10.24%), and stearic acid (8.19%), respectively. These results suggest the future utilization of myrtle fruit extracts as food additives or in chemoprevention studies.

## INTRODUCTION

Myrtaceae (*Myrtus communis* L.) is an evergreen shrub belonging to the family of Myrtaceae growing spontaneously throughout the Mediterranean area. It is a typical annual shrub of the Mediterranean countries including Turkey, Greece, Italy, Algeria, Tunisia, and Morocco. In Turkey, myrtle plants are found within the natural pine forests and riversides in the Mediterranean region, particularly in the Taurus Mountains, 500 to 600 m above sea level.[1] People living in Mediterranean region have consumed myrtle fruits, called as ‘hambeles’, ‘mersin’ or ‘murt’ in Turkish, as food, and used them for some medicinal purposes.[2] In folk medicine, a decoction of leaves and fruits or infusion of myrtle are used for stomachic, hypoglycemic, cough and oral diseases, antimicrobic, for constipation, appetizing, antihemorrhagic and externally for wound healing.[3,4] The oils of the fruits are used both in the flavor and fragrance industries.[2,5] The fruits are very astringent and are used as a condiment, as a substitute for pepper, and are considered a rich source of tannins.[6] The plant contains many biologically active compounds such as fibers, sugars, and antioxidants.[2,7]

Recent studies have focused on the healthy functions of aromatic and medicinal plants, which have antioxidant, antimicrobial, and mutagen properties.[8] Dietary intake of antioxidant compounds is important for health.[9] Although there are some synthetic antioxidants such as butylated hydroxytoluene (BHT) and butylated hydroxyanisole (BHA), which are commonly used in processed foods, it has been reported that these compounds have some side effects.[10]

Another healthy function of aromatic and medicinal plants is their essential fatty acid composition, which humans cannot synthesize, and must be obtained through diet. Essential fatty acids are necessary for the formation of healthy cell membranes and the proper development and functioning of the brain and nervous system.[11]

There is little information with regard to the antioxidant activity and fatty acid content of myrtle accessions from different regions of the world. Thus far there have been few attempts to study the fatty acid content in the fruits of myrtle, in Turkey.[12] Moreover, little information has been published with regard to the antioxidant activity of myrtle. In these studies, generally cultivated plants were used. The fatty acid content and antioxidant activities of wild growing myrtle fruits have not been reported in detail. In the present work, we report the investigation on bioactive compounds, such as, fatty acids, TP, and antioxidant activities of myrtle, to exploit its potential as a food and natural preservative.

## MATERIALS AND METHODS

### Chemicals

All the chemicals used in this study were purchased from Sigma (USA). The chemicals were of analytical grade.

### Plant materials

The fruits of wild growing myrtle were harvested manually from eight accessions found in Hatay, situated in the Mediterranean region of Turkey. The fruit colors of the accessions were white except 31-04, which was dark blue. Identification of plants was carried out by Dr. Meryem Sengul (Department of Botany, Faculty of Science, Ataturk University). A voucher specimen has been kept in Horticulture Department of the Ataturk University for future reference. The fruits were packed in a portable refrigerator during transportation to the laboratory (2-3 hours). The air-dried and powdered plant materials (100 g) were extracted in a soxhlet with methanol at 60°C for six hours. The extract was then filtered and concentrated *in vacuo* at 45°C. Finally, the extracts were kept in the dark at 4°C until tested.

The fatty acid composition was analyzed by Gas Chromatograph (GC) Clarus 500 with an autosampler (Perkin Elmer, USA) equipped with a flame ionization detector and a fused silica capillary SGE column (30 m × 0.32 mm, ID × 0.25 μm, BP20 0.25 UM, USA). The oven temperature was 140°C, held for five minutes, raised to 200°C at a rate of 4°C/minute and to 220°C at a rate of 1°C/minute, while the injector and the detector temperature were set at 220°C and 280°C, respectively. The sample size was 1 μl and the carrier gas was controlled at 16 ps. The split ratio was 1:100. Fatty acids were identified by comparing the retention times of the Fatty Acid Methyl Ester (FAME) with the standard component FAME mixture (Supelco). Triplicate GC analyses were performed and the results were expressed in GC area percent as a mean value and ± standard deviation.

The hydrogen atom or electron donation ability of the corresponding extracts and some pure compounds was measured from the bleaching of the purple-colored methanol solution of diphenylpicrylhydrazyl (DPPH). This spectrophotometric assay uses stable radical DPPH as a reagent (Sigma-Aldrich).[13] Various concentrations of extracts, of 100 μl, in methanol, were added to 5 ml of 0.004% methanol solution of DPPH. After a 30-minute incubation period at room temperature the absorbance was read against a blank, at 517 nm. Inhibition-free radical DPPH in percent (I%) was calculated as follows: $\text{I\%}=\left({\text{A}}_{\text{blank}}-{\text{A}}_{\text{sample/}}{\text{A}}_{\text{blank}}\right)100$

where A_blank_ is the absorbance of the control reaction (containing all reagents except the test compound), and A_sample_ is the absorbance of the test compound. The extract concentration providing 50% inhibition (IC50) was calculated from the graph-plotted inhibition percentage against extract concentration. Tests were carried out in triplicate and α-tocopherol was used as a positive control. In this assay, the antioxidant capacity was determined by measuring the inhibition of the volatile organic compounds and the conjugated diene hydroperoxides arising from linoleic acid oxidation.[14] A stock solution of β-carotene/linoleic acid (Sigma-Aldrich) was prepared as follows. First, 0.5 mg of β-carotene was dissolved in 1 ml of chloroform (HPLC grade), and then 25 μl of linoleic acid and 200 mg of Tween 40 (Merck) were added. The chloroform was subsequently evaporated using a vacuum evaporator. Next, 100 ml of distilled water saturated with oxygen (30 minutes at 100 ml/minute) was added with vigorous shaking. Aliquots (2.5 ml) of this reaction mixture were transferred to test tubes, and 350 μl portions of the extracts (2 g/l in methanol) were added before incubating for 48 hours at room temperature. The same procedure was repeated with α-tocopherol at the same concentration and a blank containing only 350 μl of ethanol. After the incubation period the absorbance of the mixtures was measured at 490 nm. The antioxidant capacities of the samples were compared with those of α-tocopherol and the blank.

The TP content of myrtle fruits was evaluated by employing the literature methods involving the Folin-Ciocalteu reagent and Gallic acid as a standard.[15] The extract solution (0.1 ml) containing 1000 μg extract was taken in a volumetric flask, and 46 ml of distilled water and 1 ml of Folin-Ciocalteu reagent were added and flask was shaken thoroughly. After three minutes, 2% Na_2_CO_3_ was added to 3 ml of the solution and the mixture was allowed to stand for two hours with intermittent shaking. Absorbance was measured at 760 nm. The same procedure was repeated for all standard gallic acid solutions (0-1000 mg/0.1 ml) and a standard curve was obtained.

The experiment was a completely randomized design with four replications. The data were subjected to analysis of variance (ANOVA) and the means were separated by the Duncan multiple range test at *P* < 0.05 significant level.

## RESULTS AND DISCUSSION

Fatty acid analysis has shown that the myrtle fruits studied contained 14 fatty acids [Table 1]. Oleic acid was found to be the dominant fatty acid (67.07%) followed by palmitic acid (10.24%) and stearic acid (8.19%), respectively [Table 1]. The total peak areas of the mentioned fatty acids were between 94.74-98.87% in accessions and 73.68% of these were unsaturated and 19.97% were saturated, respectively [Table 1]. The results of the present study also indicated that the fruits of myrtle are rich sources of essential fatty acids (18:2). 31-04 had a dark fruit color and several fatty acids that were not present in the other white-fruited accessions. It is possible that there is an association between fruit color and fatty acid composition.

**Table 1:** Fatty acid content (%) of fruits (mesocarp) of eight *Myrtus communis* accessions sampled from Hatay, Turkey

Common name	Molecular name	Accession								Mean
			
		31-01	31-02	31-03	31-04	31-05	31-06	31-07	31-08	
Pelargonic	9:0	-	-	-	0.24	-	-	-	-	0.03
Palmitic	16:0	5.89	10.58	9.84	11.25	8.18	10.58	14.11	11.46	10.24
Stearic	18:0	15.06	8.12	8.10	10.60	5.97	7.53	9.31	11.00	8.19
Oleic	18:1	62.13	74.66	73.22	67.77	60.07	72.30	66.70	71.71	67.07
Linoleic	18:2	12.03	02	2.70	2.96	11.16	3.28	2.47	0.64	4.78
Nonadecanoic	19:0	-	-	-	0.81	-	-	-	-	0.10
Arachidic	20:0	2.04	0.91	0.54	0.90	1.96	0.59	0.68	0.66	1.04
	20:1	-	0.34	0.18	0.67	1.06	0.21	0.88	0.23	0.45
Eicosadienoic	20:2	-	-	-	0.07	-	-	-	-	0.01
Heneicosanoic	21:0	-	-	-	0.43	-	-	-	-	0.05
	21:1	1.09	-	0.21	1.18	1.75	0.61	0.37	0.47	0.71
Behenic	22:0	-	0.93	0.34	-	1.27	-	-	-	0.31
Lignoceric	24:0	-	-	-	0.03	-	-	-	-	0.01
Nervonic	24:1	-	0.31	-	-	3.32	0.91	0.67	-	0.66
Total peak area		98.24	98.87	95.13	96.91	94.74	96.01	95.19	96.17	

-: Not detected

It was previously reported that the main fatty acids in the fruits of myrtle were oleic and palmitic acids,[12] which supported our findings. However, Cakir[12] also reported the presence of myristic and lauric acids in myrtle fruits. In our study we did not detect these fatty acids.

The free radical-scavenging activities of the methanol extracts, measured as a decolorizing activity following the trapping of the unpaired electron of DPPH, are shown in Table 2. The free radical scavenging activities were found between 2.34 (31-01) and 8.24 μg/ml (31-07). The highest free radical scavenging activity was determined in 31-01 (2.34 μg/ml). The IC50 values of the other accessions were also close to this accession and there were no statistical differences between the accessions [Table 2]. This result indicated that the extracts of myrtle fruit were extremely potent radical scavengers as also electron donors. Therefore, the extract could also react with free radicals, converting them to more stable products and terminating the radical chain reaction. This might also be important in protecting cellular DNA, lipids, and proteins from free radical damage. The results also indicated that the aqueous extracts showed considerable antioxidant activity, as measured by their capacity to scavenge the stable-free radical DPPH. As for as our literature survey, we could reach only one report that showed that the IC50 values of the methanol extract of myrtle fruit, sampled from Tunisia, was 6.5 μg/ml,[7] which supported our results.

**Table 2:** Total phenolic content, free radical scavenging capacities of the methanol extracts (100 μg/ ml) measured in DPPH assay, and inhibition ratio of the linoleic acid oxidation by the extracts *Myrtus communis* accessions sampled from Hatay, Turkey

Accession	β-carotenelinoleic acid assay (%)	*IC*_50_ (μg/ml)	Total phenolic content (μg GAE/mg dry weight)
31-01	91.65 ± 1.97^ab^	2.34 ± 0.39^NS^	74.44 ± 1.36^b^
31-02	81.07 ± 2.07^ab^	4.16 ± 0.18	56.00 ± 1.71^d^
31-03	88.64 ± 2.61^ab^	7.05 ± 0.32	51.31 ± 2.08^de^
31-04	88.56 ± 3.66^ab^	4.22 ± 0.17	44.41 ± 1.60^e^
31-05	88.31 ± 2.88^ab^	3.58 ± 0.43	88.56 ± 2.02^a^
31-06	89.80 ± 3.01^ab^	2.96 ± 0.12	56.30 ± 1.71^d^
31-07	74.53 ± 4.88^b^	8.24 ± 0.87	61.00 ± 1.55^c^
31-08	86.83 ± 2.67^ab^	3.82 ± 0.09	71.31 ± 2.11^bc^
α-tocopherol	96.31 ± 3.18^a^	2.09 ± 0.03	

*The a, b, c or ab in the same column, are results of the statistical analysis and show that there are significant differences among accessions on total phenolic content, free radical scavenging capacity, and inhibition ratio at *P* < 0.05 statistical level.

NS : Non significant

In the β-carotene/linoleic acid assay, there were statistical differences between the extracts of accessions and also accessions and α-tocopherol [Table 2]. The inhibition ratio was 96.83% in α-tocopherol followed by 31-01 (91.65%), 31-06 (89.80%), and 31-03 (88.64%), respectively. This result suggested that in the β-carotene/linoleic acid assay, oxidation of linoleic acid was effectively inhibited by the myrtle fruit extract. Therefore, it can be concluded that myrtle fruits have a strong antioxidant capacity and can be important both in the human diet and for food safety, when mixed with other foods. In the previous studies conducted on different cultivars of myrtle, the antioxidant activity was found to be between 76.7 and 99.0% in aqueous, methanol, and α-tocopherol extracts, respectively, which was very close to our results.

The TP of accessions was between 44.41 and 88.56 μg GAE/mg dry weight equivalents of phenolic compounds in the extract. There were statistical differences among accessions in terms of TP [*P* < 0.05, Table 2]. The antioxidative effectiveness in natural sources was reported to be mostly due to TP.[16] The results for TP in fruit extracts clearly outline the rich phenolic sources. The variability among accessions could be due to the plants used, environmental factors, and collection period. Phenolic compounds are the antioxidants that contribute to the high antioxidant capacity observed in edible plants.[17]

The chemical composition of myrtle leaf extracts have been studied previously and the results have shown and determined that flavonoids, coumarins, and tannins are the major compounds.[7] Flavonoids are the most probable candidates among the compounds, known to be present in the methanol extracts, which prevent oxidative lesions.[18]

## CONCLUSION

As a conclusion from these results, it can be suggested that the consumption of myrtle can probably offer some dietary benefits, as they contain antioxidant constituents, which can protect against lipid peroxidation and can scavenge free radicals. This report further suggests that myrtle may have beneficial chemopreventive effects in addition to providing potential new sources of natural antioxidants as well as genes, and also for use as food and for medicinal purposes. However, further studies to fractionate the active extracts, to identify the active compounds, and to determine the exact mechanism of action, are required. Moreover, the sampling date and phenotype factors should be considered in future efforts, to improve and domesticate myrtle as a major agricultural crop.
